# Role of interfaces on the stability and electrical properties of Ge_2_Sb_2_Te_5_ crystalline structures

**DOI:** 10.1038/s41598-017-02710-3

**Published:** 2017-06-01

**Authors:** A. M. Mio, S. M. S. Privitera, V. Bragaglia, F. Arciprete, S. Cecchi, G. Litrico, C. Persch, R. Calarco, E. Rimini

**Affiliations:** 1 Institute for Microelectronics and Microsystems (IMM), Consiglio Nazionale delle Ricerche (CNR), VIII Strada 5, 95121 Catania, Italy; 2 Paul-Drude-Institut für Festkörperelektronik, Hausvogteiplatz 5-7, 10117 Berlin, Germany; 3 Dipartimento di Fisica, Università di Roma “Tor Vergata”, Via della Ricerca Scientifica 1, I-00133 Rome, Italy; 4 I. Physikalisches Institut (IA), RWTH Aachen University, Sommerfeldstraße 14, 52074 Aachen, Germany

## Abstract

GeSbTe-based materials exhibit multiple crystalline phases, from disordered rocksalt, to rocksalt with ordered vacancy layers, and to the stable trigonal phase. In this paper we investigate the role of the interfaces on the structural and electrical properties of Ge_2_Sb_2_Te_5_. We find that the site of nucleation of the metastable rocksalt phase is crucial in determining the evolution towards vacancy ordering and the stable phase. By properly choosing the substrate and the capping layers, nucleation sites engineering can be obtained, thus promoting or preventing the vacancy ordering in the rocksalt structure or the conversion into the trigonal phase. The vacancy ordering occurs at lower annealing temperatures (170 °C) for films deposited in the amorphous phase on silicon (111), compared to the case of SiO_2_ substrate (200 °C), or in presence of a capping layer (330 °C). The mechanisms governing the nucleation have been explained in terms of interfacial energies. Resistance variations of about one order of magnitude have been measured upon transition from the disordered to the ordered rocksalt structure and then to the trigonal phase. The possibility to control the formation of the crystalline phases characterized by marked resistivity contrast is of fundamental relevance for the development of multilevel phase change data storage.

## Introduction

In many areas of science and technology, such as production of semiconductors, but also of pharmaceuticals, as well as formation of biominerals, it is essential to control crystallization processes. These usually occur via nucleation and growth and, in most practical circumstances, crystallization starts with heterogeneous nucleation at a foreign surface^1^. Despite its widespread occurrence, mechanistic understanding of the role of a surface in heterogeneous nucleation is limited. However, to control crystallization, the contribution of different surface properties to the effectiveness of a surface in inducing nucleation must be elucidated. Indeed the presence of interfaces can modify the nucleation process through various means, such as via favourable interactions with the crystallizing material and lattice match between the substrate and the compound to be crystallized.

Here we study the effect of the interfaces on the crystallization of the metastable rocksalt phase in amorphous GeSbTe (GST) thin films, to explore the correlation between the produced microstructure and the subsequent path followed for the conversion from the rocksalt phase to the stable phase, with trigonal structure. Thanks to the ability to rapidly switch between two phases with high electrical and optical contrast, GST alloys belonging to the GeTe-Sb_2_Te_3_ pseudo-binary line are the optimal candidate for non volatile phase change memories^2–4^ as well as for electronic displays^5^ and ovonic threshold switches^2, 6^. The phases used as logic states are usually the amorphous and the metastable phase, with rocksalt structure. It is well assessed in literature that in the rocksalt structure Te occupies the anion sites and the cation sites are randomly occupied by Ge, Sb and vacancies^7^. Recently it has been shown that vacancy ordering can be induced in the metastable phase^8–10^, giving rise to an ordered rocksalt phase in which the electronic transport is modified, changing from an insulating transport, typical of the rocksalt phase with random vacancy distribution, to a metallic behaviour^8^. Currently, multi-level storage^11, 12^ has been realized by controlling the fraction of the crystalline^11^ or amorphous^13^ regions within a cell. The vacancy ordering could be used for multi level bit storage by employing amorphous, disordered rocksalt and ordered rocksalt or trigonal, to make three different logic states exhibiting contrast in the resistance value. However, in order to develop devices according to this new approach, a detailed understanding of the growing conditions and/or interfaces that can affect the vacancy ordering, as well as the relationship between the vacancy ordering and the electrical properties is required.

In this paper we show that the nucleation of the metastable phase plays a relevant role, since both the process of vacancy ordering and the electrical conductivity are extremely sensitive to the interfaces and to the microstructure of the metastable rocksalt phase. We also show that three clearly distinct resistivity levels are associated to the crystalline structure with different degrees of order.

## Results and Discussion

### Surface Effects on the nucleation of the rocksalt phase

The phase change films were deposited in the amorphous phase on different substrates: Si (111) or SiO_2_ deposited on a Si (100) wafer. The films have been then converted into the metastable rock-salt structure by *ex-situ* thermal annealing. The formation of the metastable structure, as well as the vacancy ordering can be followed by XRD and Raman spectroscopy.

**Figure 1:**
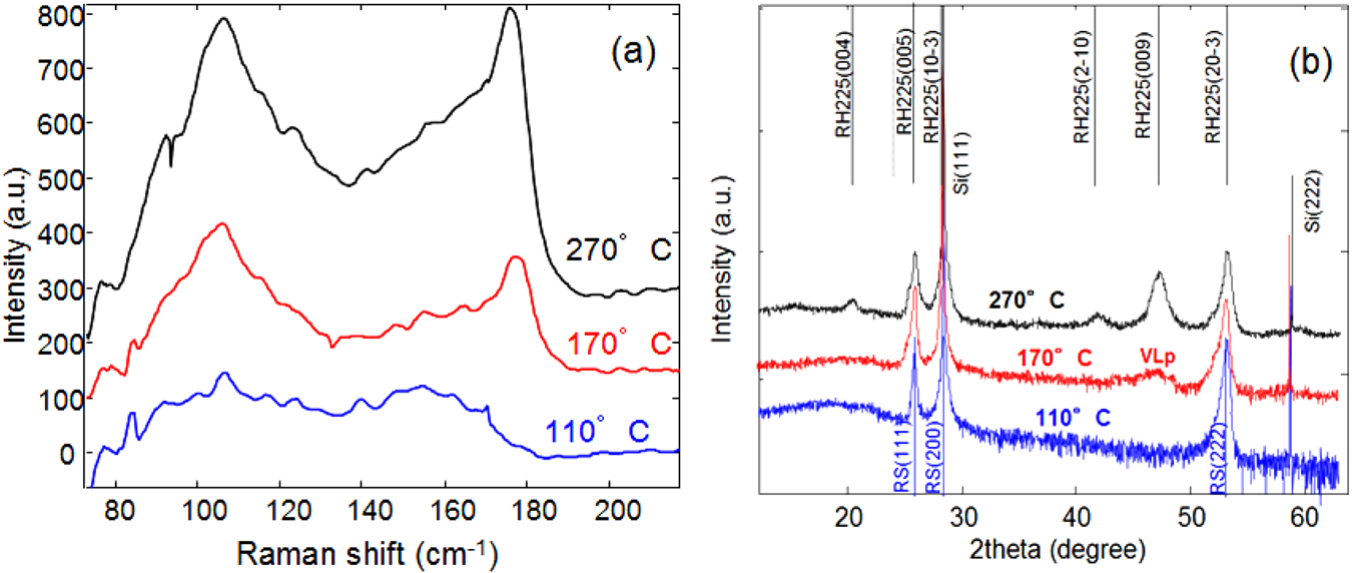
(**a**) Raman spectrum of GST samples deposited in the amorphous phase on Si (111) and annealed at different temperatures. (**b**) X-ray diffraction pattern of the same samples acquired in ω-2θ scan.

**Figure 2:**
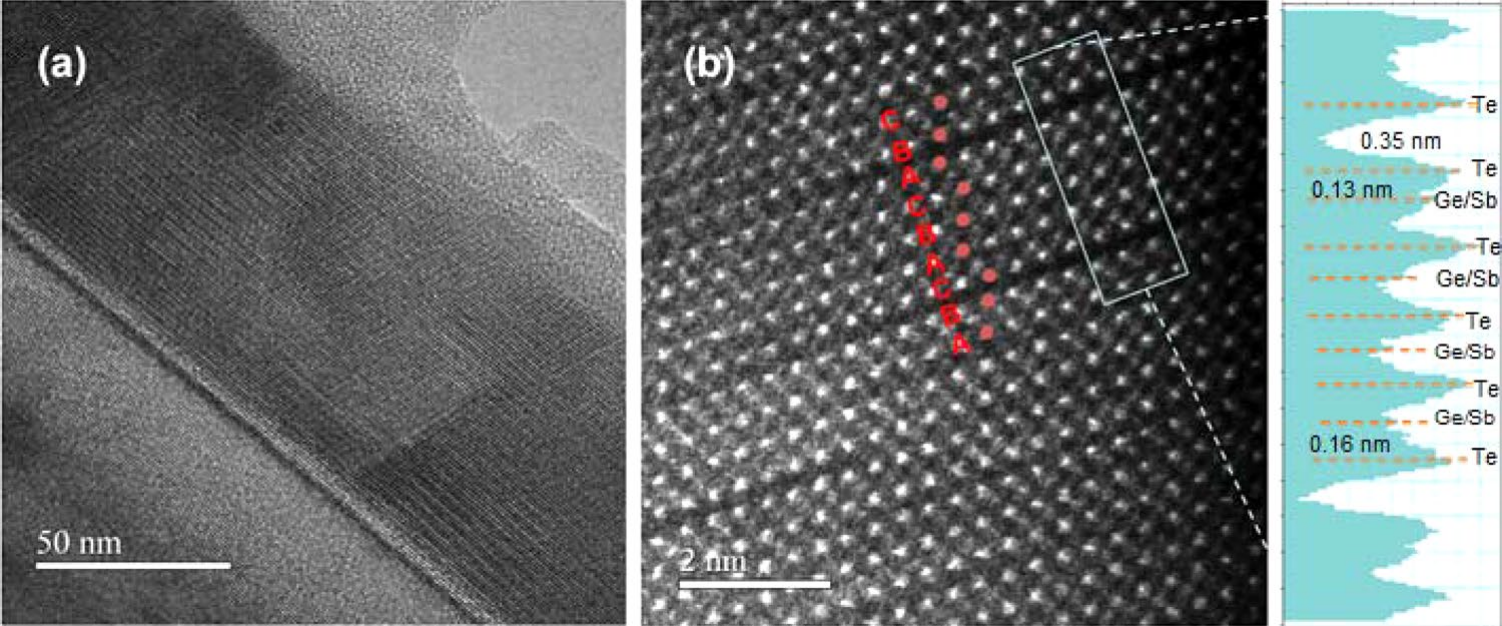
HAADF STEM micrograph of the GST on Si (111) annealed at 170 °C. (**a**) The texturing of the film is visible. In (**b**) the dark regions are vacancy layers with missing atoms, ordered according to a cubic stacking. The intensity profile on the right indicates a reduced distance between Te planes close to the vacancy layer, and the adjacent cationic plane.

Figure 1(a) and (b) show the Raman spectra and the XRD pattern of amorphous GST deposited on Si (111) and annealed at different temperatures. The crystalline phase formed at the lowest temperature (110 °C) is the disordered rocksalt phase, characterized by a large peak in the Raman spectrum at about 155 cm^−1^. By annealing at 170 °C the Raman spectrum appears largely modified, with the peak at 155 cm^−1^ completely absent and the appearance of a sharp peak at 178 cm^−1^. This peak, typical of the trigonal phase, is associated to the A_1g_ Raman mode^14, 15^ and it is also observed in the sample annealed at 270 °C. XRD spectra acquired in Bragg-Brentano configuration, shown in Fig. 1(b), reveal that the sample annealed at highest temperature is indeed in the trigonal phase and it is highly textured with the {0001} planes parallel to the surface. Instead the sample annealed at 170 °C exhibits the diffraction pattern typical of the cubic phase, but with a small and broad peak at 47°, that has been ascribed to the presence of ordered vacancy layers^8^. Figure 2(a) shows a TEM micrograph of the film on Si (111) after annealing at 170 °C. It is polycrystalline, but highly textured with (111) planes parallel to the surface.

Since the A_1g_ Raman peak is related to the oscillation of Te atoms close to the van der Waals gaps^15, 16^, the presence of such a peak in a sample with cubic structure and ordered vacancy layers could sound unexpected. However, we find here that this oscillation is representative not simply of the ordering of the vacancy layers, but also of the complete modification of the Te bonds. Indeed, in the sample annealed at 170 °C the stacking of the planes is that of the rocksalt phase (ABCABC), as shown by the red spots in the TEM micrograph of Fig. 2(b). The width of the vacancy layers is double than the distance between two adjacent planes in the rocksalt structure (indicating that it is a vacancy layer, not a van der Waals gap). However, as shown in the intensity profile, the distance between the cationic planes adjacent to the Te plane at the vacancy layer is reduced to about 0.14 nm. Such a value is less than the distance (0.17 nm) between two planes in the disordered rocksalt structure, and suggests a situation more similar to the trigonal phase, therefore indicating that a modification of the Te bonds has occurred.

**Figure 3:**
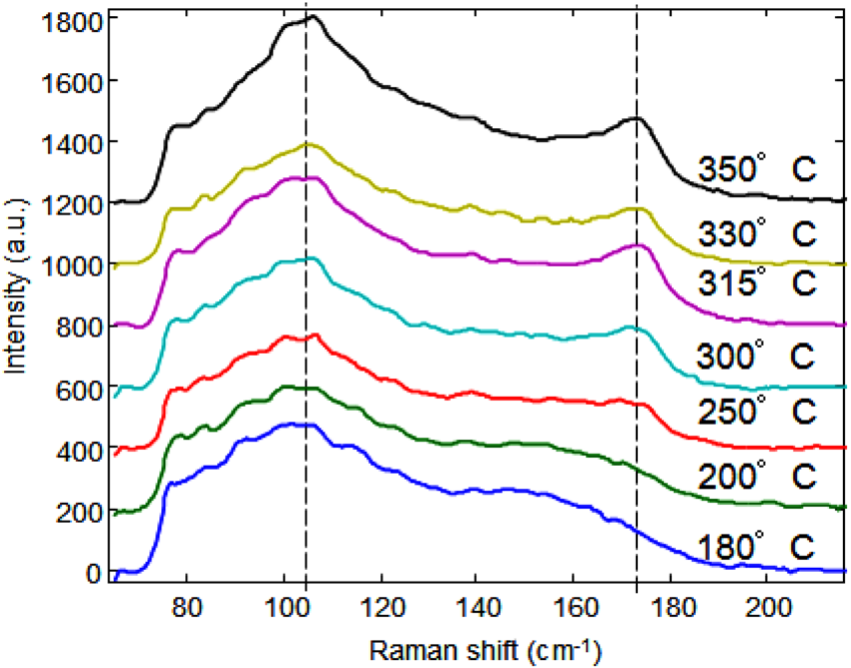
Raman spectra of GST deposited amorphous on SiO_2_, covered with a ZnS:SiO_2_ capping layer, and then annealed at different temperatures.

The microstructure of the GST film deposited on SiO_2_ appears very different. In this case annealing at 110 °C for 1 h is not sufficient to complete the crystallization of the rocksalt phase. Figure 3 shows the Raman spectra acquired for GST films on SiO_2_, covered also by a thin ZnS:SiO_2_ cap layer. Films annealed up to 200 °C exhibit the typical spectrum of the disordered rock-salt phase, with a peak at about 155 cm^−1^. An intermediate situation is represented by the sample annealed at 250 °C, in which the peak at 155 cm^−1^, typical of the disordered rocksalt phase, disappears. Only above 250 °C the A_g1_ peak is clearly detectable. At higher temperature, up to 350 °C the contribution at about 155 cm^−1^ continuous to decrease and, according to previous studies on the effect of ordering on the trigonal structure, this could correspond to the progressive increase of Sb occupation at the cationic planes close to Te atoms at the van der Waals gaps^17, 18^.

**Figure 4:**
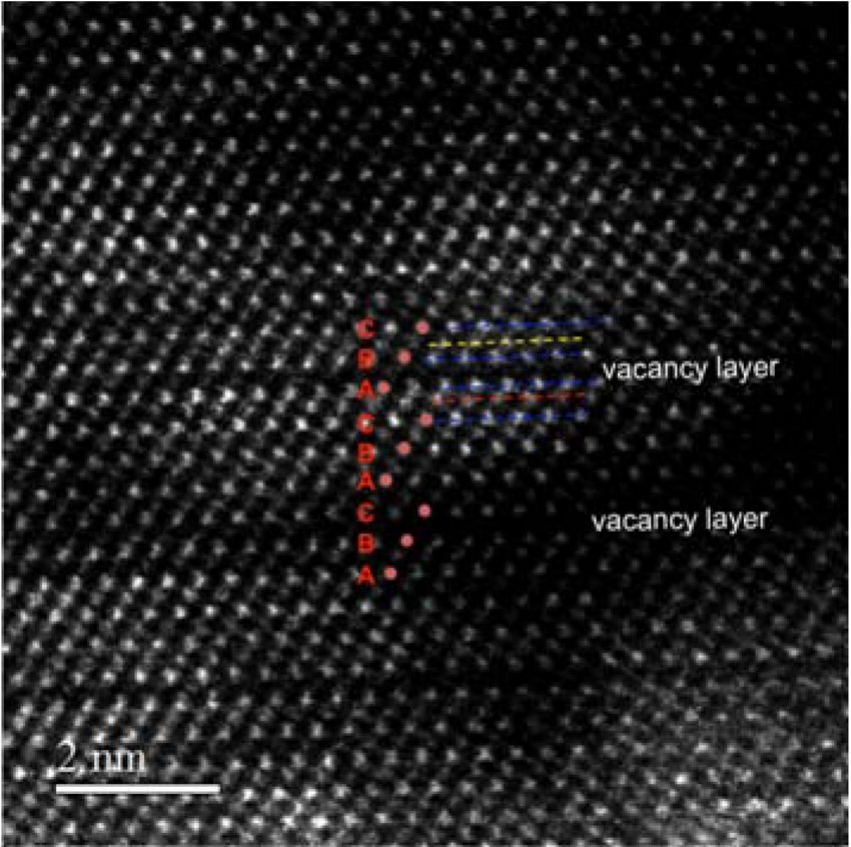
Dark Field STEM micrograph of the GST on SiO_2_ annealed at 250 °C, acquired with an inner detector semiangle of 42 mrad. The stacking is that of the rocksalt phase, with ordered vacancy layers, as shown by the red spots. The distance between Te layers close to the vacancy layer (blue dashed lines) and the first cation plane is lowered only in some cases (red dashed lines).

STEM analysis in dark field of the sample annealed at 250 °C, shown in Fig. 4, reveals that it has a rocksalt structure with ordered vacancy layers. According to the STEM intensity, and in very good agreement with the Raman spectrum, the bonding of the Te atoms close to the vacancy layers is not completely modified, indicating the vacancy layers are still not completely empty.

It is known in literature^19, 20^ that in GST films deposited on SiO_2_ substrate the nucleation of the rock-salt phase is heterogeneous, as indicated by the low activation energy barrier for nucleation of a critical nucleus ΔG* (0.3 eV). It has been also shown by *in situ* TEM analyses that the nucleation starts at the amorphous film top surface^21, 22^ and it is followed by a second heterogeneous nucleation regime, with the formation of grains also at the GST/SiO_2_ interface. In the case of GST deposited on Si (111), the presence of a strong texturization with the (111) plane parallel to the surface, clearly indicates that the nucleation of the GST rock-salt structure has occurred preferentially at the interface with the Si substrate. In order to avoid the competition between different nucleation seeds, here we have intentionally left the GST film uncovered, and performed the annealing for crystallization in vacuum. In this way we have obtained nucleation site control and engineering^23^.

In the classical nucleation theory the most important parameter governing the heterogeneous nucleation is the contact angle θ. Since ΔG*_het_ = f(θ) ΔG*_hom_ where ΔG*_het_ is the free energy barrier for heterogeneous nucleation, ΔG*_hom_ for homogeneous nucleation, and f(θ) = 1/4 (2−3cos θ + cos^3^θ) is the wetting function.

**Figure 5:**
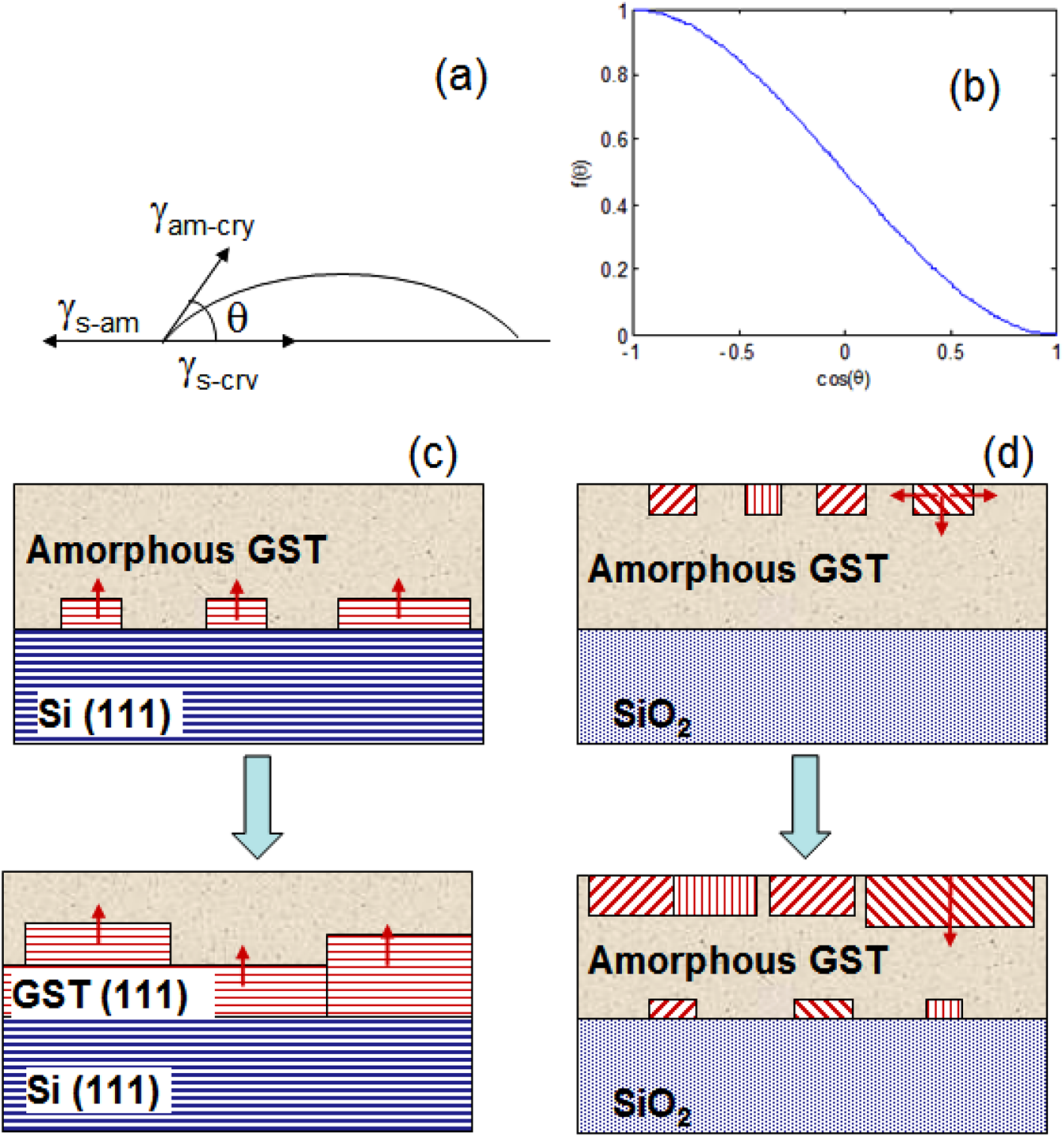
(**a**) Schematic of the surface energy and the contact angle; (**b**) f(θ) versus cos(θ). (**c**) and (**d**) Schematic of the heterogeneous nucleation sites and their evolution in the case of Si(111) and SiO_2_ substrates, respectively.

Being cos(θ) = (γ_s−am_ − γ_s−cry_)/γ_am−cry_ with γ_s−am_ surface energy between substrate and GST amorphous, γ_s−cry_ substrate-GST crystalline and γ_am−cry_ between GST amorphous-crystal, as schematically drawn in Fig. 5(a).

Therefore the smaller θ is, the greater the affinity between the nucleus and the substrate in the crystallizing medium and the lower the free energy barrier for heterogeneous nucleation^24^.

Figure 5(b) shows the function f(θ) determining the reduction of the nucleation barrier due to the heterogeneous nucleation, as a function of the contact angle. In Fig. 5(c) and (d) the heterogeneous nucleation sites and their evolution as a function of temperature are shown for Si(111) and SiO_2_ substrate, respectively.

According to our observation we can roughly conclude that:

$$({{\rm{\gamma }}}_{{\rm{Si}}(111)-{\rm{am}}}-{{\rm{\gamma }}}_{{\rm{Si}}(111)-{\rm{cry}}}) < ({{\rm{\gamma }}}_{{\rm{air}}-{\rm{am}}}-{{\rm{\gamma }}}_{{\rm{air}}-{\rm{cry}}}) < ({{\rm{\gamma }}}_{{\rm{SiO}}2-{\rm{am}}}-{{\rm{\gamma }}}_{{\rm{SiO}}2-{\rm{cry}}}).$$

However, this approach is limited by the assumption of the classical nucleation theory and it assumes that the nucleus is cap shaped rather than multifaceted, since the microscopic interfacial free energies are assumed to be the same. In the reality the surface energy of the crystal has a pronounced dependence on the orientation of the surface. For a grain with rock-salt structure, based on broken bonds model, the surface with lower surface energy is the (100). The (111) surface is instead characterized by the highest number of broken bonds and it has therefore a higher surface energy.

### Surface Effects on vacancy ordering and conversion to the trigonal phase

Engineering of the nucleation sites does not only affect the microstructure and the surface orientation of the rocksalt phase, but it has also a strong effect on the ordering of the vacancy layers. This usually occurs at temperatures in the range between the (disordered) rock-salt crystallization temperature and the temperature at which transition to the trigonal phase occurs.

**Figure 6:**
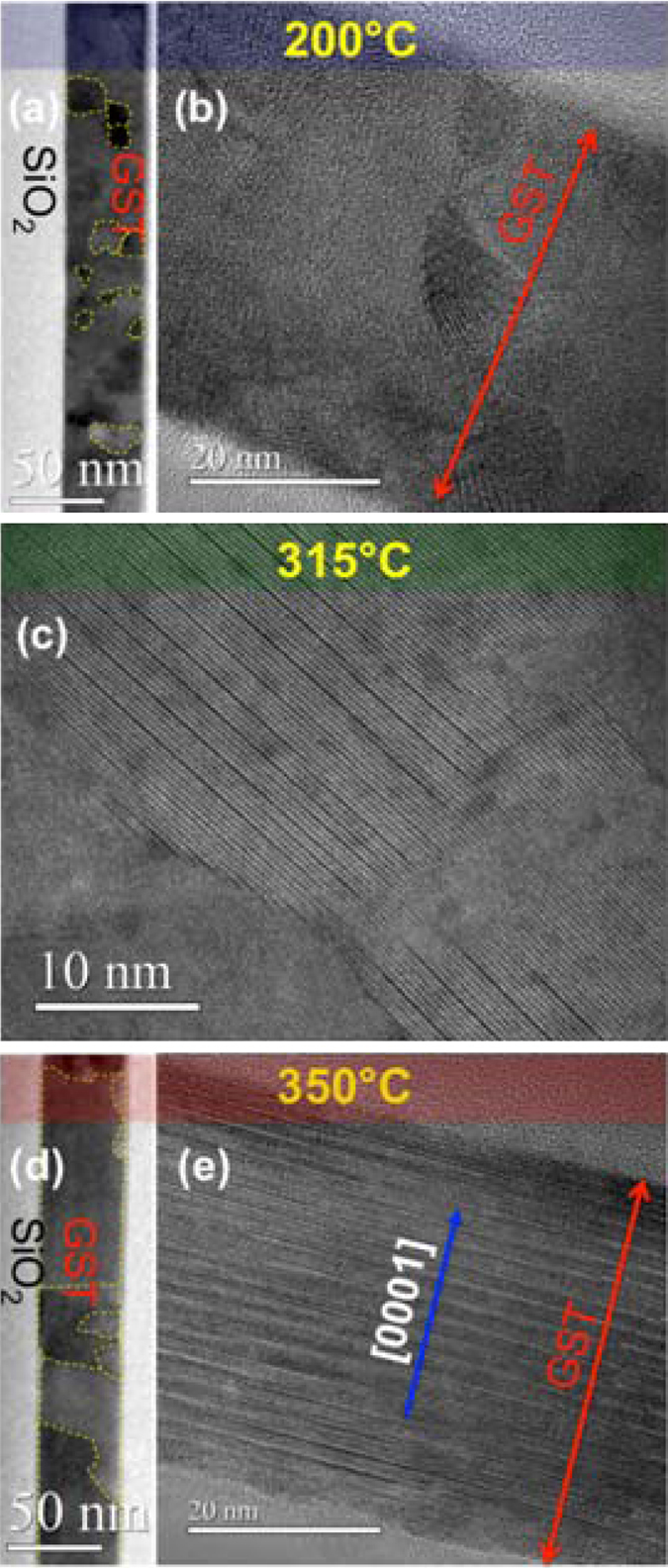
Evolution of the crystalline phases as a function of temperature for GST film on SiO_2_. (**a**) and (**b**) shows a TEM micrograph of a sample annealed at 200 °C. Fine randomly oriented grains with rocksalt structure are visible and grain boundaries are underlined in (**a**) by dashed yellow lines. (**c**) GST annealed at 315 °C, exhibiting both trigonal and rocksalt phase, with ordered vacancy layers or random vacancies. (**d**) and (**e**) shows the sample in the trigonal structure annealed at 350 °C. The presence of large grains (>50 nm) is shown by yellow dashed lines, indicating the grain boundaries in (**d**). {0001} planes parallel to the surface are preferentially observed.

Figure 6 shows the microstructure evolution of the film deposited on SiO_2_ upon annealing at different temperatures, from 200 °C to 350 °C. At 200 °C the material is in the disordered rocksalt phase and exhibits randomly oriented fine grains (Fig. 6(a) and (b)). Upon annealing at 315 °C, as shown in Fig. 6(c), both the rocksalt with ordered or disordered vacancies, as well as the trigonal phase can be detected.

**Figure 7:**
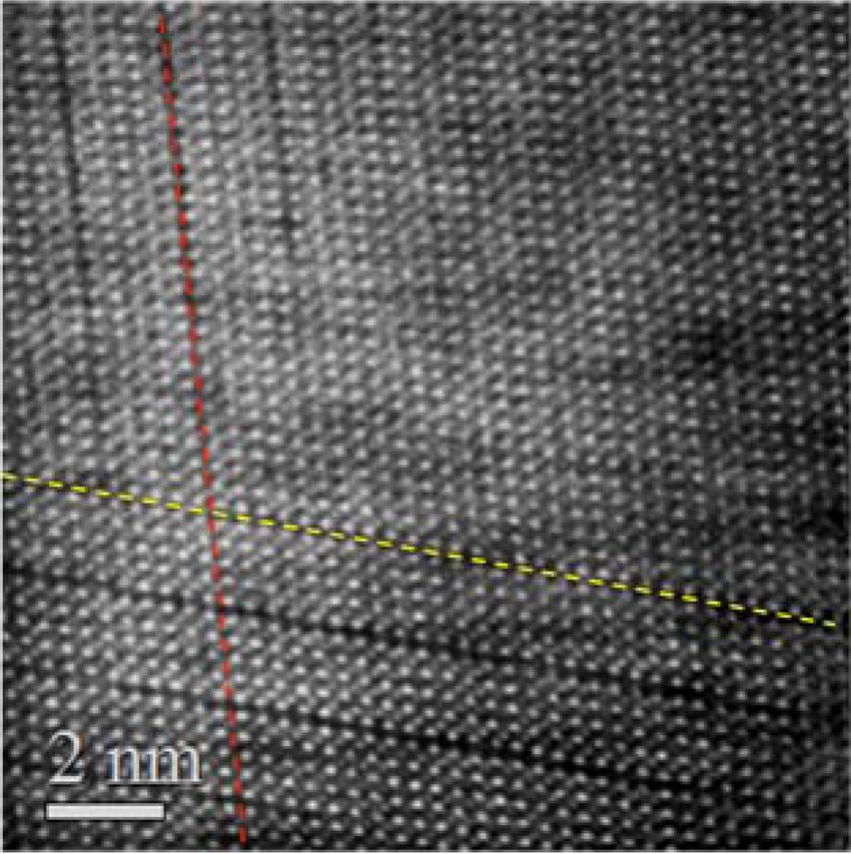
HAADF STEM micrograph of a sputtered sample, annealed at 315 °C, exhibiting rocksalt structure and ordered vacancy layers on two different {111} planes, indicated with red and yellow dashed lines.

The vacancy ordering process in the rocksalt phase occurs at the {111} planes^25, 26^. In a single grain these planes form a tetrahedral. The intersection of two of such {111} planes at an angle of 70.5° is shown in the TEM cross section micrograph of Fig. 7. The situation may be similar to the formation of stacking fault tetrahedra in metals with face centered cubic structure under cold work plastic deformation, quenching experiments from temperatures close to the melting point or under irradiation^27, 28^.

However, only one of the {111} planes with ordered vacancies would be useful for the conversion of the materials into the trigonal structure. The other three planes should be annealed out. Figure 6(c) shows that grains with different orientations start to align their vacancy layers and following this process much larger grains with trigonal structure will be obtained. This process is completed upon annealing at 350 °C, after which the film is completely converted into the trigonal phase. Although polycrystalline, the film exhibits a marked texturing, with the {0001} planes parallel to the surface. Figure 6(d) shows that in the trigonal phase the grain size is larger and Fig. 6(e) is representative of typical grain orientation.

The theory for the equilibrium shape of crystals determined by the difference of surface energy, based on broken bonds model, is again illuminating in determining the growth of trigonal structured Ge_2_Sb_2_Te_5_. As it has been shown in ref. 29, the planes which are (0001), (1–103), and (1–106) have low surface energy, with (0001) < (1–106) < (1–103). Since the surface energy depends on the number of atoms in each plane, this scheme is typical of the rhombohedral stacking. Indeed, also for trigonal GST it has been often reported a preferential orientation of the (0001) plane at the surface, even for samples deposited on SiO_2_ substrate.

Going back to the sample deposited on Si(111), we observe that it is already aligned with a (111) plane parallel to the surface, even in the rocksalt phase. This situation facilitates the vacancy ordering, which occurs at much lower temperature (170 °C), compared to the randomly oriented sample (250 °C).

Considering the equivalency between the {111} planes of the rocksalt structure and the {0001} of the trigonal one, it is clear that the presence of {111} rocksalt planes parallel to the surface may reduce the atomic movements required to reach the situation corresponding to the minimum surface energy, i.e. with the trigonal (0001) plane at the surface, thus facilitating the conversion to the trigonal phase. Texturization starting from Si (111) is therefore also advantageous for the vacancy ordering and the transition to the trigonal structure.

In the case of a silicon oxide substrate, instead, several {111} planes with ordered vacancies, not necessarily parallel to the surface, may form. In order to reach the stable configuration with trigonal (0001) planes at the surface more atomic planes need to be modified.

### Surface effect on the electrical properties

The electrical properties of GST are strongly dependent on the ordering of the crystalline structure and this fact is crucial for developing phase change memories that can be based on the ordering of the structure, such as for example, interfacial phase change memories or for multi-level storage.

The trigonal phase is known to have a metallic behaviour while the disordered rocksalt structure is an insulator^30^. It has been shown that a transition from an insulating to a metallic behaviour (MIT) may be obtained by thermal annealing either in the trigonal structure (as shown for GeSb_2_Te_4_)^30^ or in the rocksalt structure^8^. In particular it has been shown that the MIT in the rocksalt structure is due to the ordering of the vacancy layers^8^ and, on the contrary, by introducing disorder in the trigonal phase through ion irradiation, a reverse metal-to-insulator transition can be observed, driven by disordering of the vacancy layers^16^.

Here we find that the resistivity, as well as its behaviour as a function of temperature, which is modulated by the vacancy ordering and by the subsequent conversion to the trigonal structure, are strongly dependent on the interfaces.

**Figure 8:**
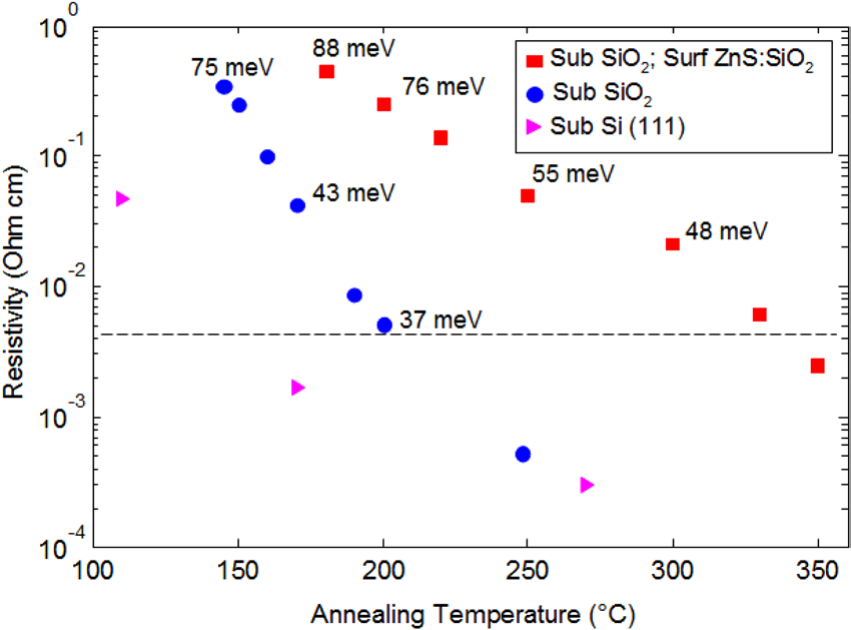
Resistivity measured at room temperature in samples prepared with different interfaces, as a function of annealing temperature. The dashed line indicates the value of 5 mOhm cm. The energy values indicated in the figure are the mobility gaps, obtained from the temperature variation of resistance in the range 40–100 °C.

Figure 8 shows the resistivity as a function of annealing temperature as measured on samples with different interfaces: on SiO_2_ without capping layer, on SiO_2_ with ZnS:SiO_2_ capping layer, and on Si(111) without capping. The sample on Si(111) exhibits the lower resistivity, probably due to the larger grain size. Ordering of vacancy layers occurs at lower temperature and induces the MIT at 170 °C. It is important to note that, although the resistance values and temperature conditions may be quite different by changing the interfaces, in all of the samples the MIT is observed to occur for resistivity around 5 mOhm cm or below, i.e. at the maximum resistivity for a metal according to the Anderson model, as reported in ref. 30. In particular, it is shown^30^ that the MIT occurs when the mobility edge crosses the Fermi level. According to this description, in films with rocksalt structure or with mixed rocksalt-trigonal phase, the activation energy governing the temperature dependence of the resistivity represents the distance between the mobility edge and the Fermi level (mobility gap). The energy values reported in Fig. 8 indicate the measured mobility gaps in the range 40–100 °C. In the case of randomly oriented rocksalt grains, obtained on a SiO_2_ substrate without capping layer, the resistance rapidly decreases as a function of the annealing temperature and the vacancy ordering occurs at about 200 °C, as indicated by the disappearing of the peak at 155 cm^−1^ in the Raman spectrum (not shown). However, the vacancy ordering, in this sample, appears to be a competing process with the formation of the trigonal phase, that is formed at about 250 °C, not very different from the case of Si(111) substrate. The mobility gap is 75 meV in the disordered rocksalt phase, formed at 140 °C, and it monotonically decreases as the degree of order increases. The MIT occurs at about 200 °C, for mobility gap lower than 30 meV, indicating the mobility edge crosses the Fermi level within KT at room temperature (≈26 meV).

In the sample with the ZnS:SiO_2_ capping layer the nucleation of the (disordered) rocksalt phase occurs at the highest temperature, as well as the vacancy ordering and the conversion to the trigonal structure. The MIT is observed at about 330 °C and then the conversion into the trigonal phase occurs at about 350 °C. These results indicate that the capping layer inhibits the nucleation at the top surface. The nucleation of the rocksalt phase therefore may occur at the interface with the SiO_2_, which is characterized by higher surface energy.

For the samples on Si (111) the mobility gap can not be evaluated in the temperature range 40–100 °C, since it is affected by the Si conductivity, as well as by the onset of crystallization, occurring at temperatures around 100 °C. Low temperature measurements^8^ show a weak temperature dependence of the resistivity even in the case of disordered rocksalt phase.

Nevertheless, in all the investigated samples, depending on the degree of ordering of the structure, three distinct resistance values can be distinguished, with a difference of about one order of magnitude, by changing from disordered to ordered rocksalt and to the trigonal phase.

## Conclusions

The role of the interfacial energy on the structure and electrical properties of Ge_2_Sb_2_Te_5_ has been investigated. We have explored the correlation between the sites of nucleation of the rocksalt phase and the subsequent path followed for the conversion into the stable phase, with trigonal structure. This work evidences how the nucleation of the metastable phase, and consequently its microstructure and grain orientation, is a crucial factor in determining the vacancy layers ordering and the evolution towards the stable trigonal phase. Decrease of about one order of magnitude in the resistivity has been observed upon vacancy layer ordering and then a further decrease has been measured upon transition to the trigonal phase. Therefore our data show that it is possible i) to obtain nucleation sites engineering by properly choosing the substrate and cap layer; ii) to clearly distinguish upon three different resistance values, characterized by different degree of structural order. The present results are very promising for the development of multi state phase change data storage.

## Methods

### Sample preparation

Ge_2_Sb_2_Te_5_ films were deposited in amorphous phase either on Si(111) or SiO_2_ substrate. Sample on Si (111) were deposited by molecular beam epitaxy (MBE) equipped with separate Ge, Sb, and Te effusion cells and then annealed *ex-situ* at several temperatures by means of rapid thermal annealing. Ge_2_Sb_2_Te_5_ films on SiO_2_ were deposited by sputtering on silicon oxide at room temperature, using a single stoichiometric target. Some samples have been capped with a 10 nm thick ZnS:SiO_2_ layer. Annealing treatments were then performed at several temperatures in the range 150 °C–350 °C for 30 min in a vacuum furnace. The film thickness was 50 nm for the sputtered films and 25 nm for the MBE films.

The specimens for TEM analysis were prepared by standard cross-sectional mechanical polishing followed by Ar^+^ ion milling at ≈100 °C, using a Gatan PIPSII system. Ion energy ranged from 2.0 keV to 0.1 keV in order to avoid sample amorphization and damaging during thinning.

### HAADF STEM

A JEOL ARM200F Cold FEG condenser Cs-corrected STEM/TEM working at 200 kV was adopted to obtain High Resolution (HR) micrographs of the sample. The HAADF STEM images were obtained with a convergence semiangle of 33 mrad, a nominal point resolution of 0.68 Å. We operated at a very high Dark Field detector inner semi-angle (83 mrad) at which the scattering cross-section is well approximated by the Rutherford formula^31^, predicting an intensity roughly proportional to Z^2^. In some cases, as indicated, we have used also 42 mrad. HAADF STEM, compared to conventional TEM, is almost free of delocalization phenomena, at this high detection semi-angle, because also of the large incoherent electron scattering.

Referring to the trigonal cell, the film was observed mainly along the < 11–20 > direction in order to directly analyze the stacking sequence of the atomic planes along the c-axis. The HAADF Micrographs were obtained using a dwell time per pixel of 40 μs and an electron beam current of about 50 pA. This low value should avoid any relevant artifact.

### Raman Spectroscopy

A Horiba Jobin Yvon HR800 system equipped with a 633 nm HeNe laser has collected Raman spectra. In order to avoid heating of the sample, the power of the laser was kept below 1 mW, with a laser spot diameter of about 4 μm. The spectral window is 320 cm^−1^ and the resolution 0.2 cm^−1^. Each spectrum has been acquired using 3 accumulations, each with collecting time of 40 s.

### X-Ray diffraction

Samples were characterized by means of *ex-situ* X-ray diffraction (XRD), utilizing a PANalytical X’ Pert PRO MRD diffractometer with Ge (220) hybrid monocromator, Employing a Cu Kα_1_ radiation (λ = 1.540598 Å). Specular ω−2ϑ scans were performed in double axes mode in order to access the growth direction of the films, in a range of 10°–110°, with a step 0.02° and integration time of 2.5 s.

### Electrical Measurements

The electrical properties have been studied by measuring the sheet resistance with a four point probe and its temperature dependence has been evaluated using a Temptronics thermal chuck, in the range from 20 °C to 100 °C. For resistance measurements a HP4156B parameter has been employed.
